# Dual-process theories beyond cognitive psychology: psychodynamic, clinical, and neurobiological perspectives

**DOI:** 10.3389/fpsyg.2026.1936753

**Published:** 2026-09-18

**Authors:** Özge Türkoğlu, Başaran Demir

**Affiliations:** 1 Department of Psychiatry, Soma State Hospital, Manisa, Türkiye; 2 Department of Psychiatry, Faculty of Medicine, Hacettepe University, Ankara, Türkiye

**Keywords:** cognitive science, dual process theories, neuroscience, primary process, psychodynamic theory, psychopathology, psychotherapy

## Abstract

Dual-process theories (DPT) have become an influential framework for understanding human cognition by distinguishing between two modes of mental processing, often described through distinctions such as automatic vs. controlled, intuitive vs. analytic, or unconscious vs. conscious. Although initially developed within cognitive psychology, this review considers DPT in relation to psychodynamic, clinical, and neurobiological perspectives to explore their complementary contributions to understanding human mental functioning. From a psychodynamic perspective, particular attention is given to primary process thinking and its potential contribution to contemporary debates concerning the nature and organization of different modes of processing. We suggest that primary process has been conceptualized in two broad ways: as a qualitatively distinct mode of processing or as a less differentiated form of a shared underlying mental organization, with potential implications for how different modes of mental processing are understood and investigated. Clinical perspectives further demonstrate the relevance of these theories across a range of phenomena—from psychosomatic symptoms to depression—as well as in clinical decision-making and psychotherapeutic change. Neurobiological findings provide insights into different aspects of dual-process distinctions and highlight the involvement of dynamically interacting brain networks rather than discrete and independent neural systems. Rather than proposing direct equivalences between constructs and findings from different fields, this review argues that DPT provides a useful conceptual framework for examining related psychological phenomena across psychodynamic, clinical, and neurobiological perspectives. In doing so, it may facilitate interdisciplinary research and contribute to a more comprehensive understanding of human mental functioning and psychopathology.

## 1 Introduction

Understanding the architecture of the human mind and the principles underlying its organization has long been a central goal of cognitive science. Among the theoretical frameworks developed to address this goal, Dual Process Theories (DPT) represent one of the most influential approaches. DPT propose that cognitive processes can generally be understood through a distinction between fast, automatic, and unconscious processes and slow, controlled, and conscious processes (Evans, 2008). Although the idea of dual modes of cognition has a long intellectual history, modern DPT emerged within cognitive psychology as a framework for explaining human cognition. Since then, they have been applied across a wide range of disciplines, including social psychology, behavioral economics, cognitive neuroscience, and learning and memory research (Evans, 2008; Kahneman, 2011; Gronchi and Giovannelli, 2018; Gawronski et al., 2024).

DPT do not constitute a single, unified theory but rather a family of theoretical frameworks that conceptualize dualities in mental functioning in different ways (Evans and Stanovich, 2013). No universally accepted terminology exists for these dual processes, and different researchers have referred to them using labels such as automatic/controlled, intuitive/analytic, and implicit/explicit (Table 1) (Evans, 2008). Nevertheless, there is broad agreement that one process is relatively fast, automatic, unconscious, and largely independent of working memory, whereas the other is relatively slow, deliberative, conscious, and dependent on working memory (Evans, 2008; Evans and Frankish, 2009). However, these characteristics should be understood as broad theoretical tendencies rather than fixed or universally shared properties, and their specific definitions and combinations vary across dual-process accounts.

**Table 1:** Common distinctions between Type 1 and Type 2 processing proposed in the dual-process literature.

Type 1	Type 2	Selected references
Automatic	Controlled	(Schneider and Shiffrin, 1977)
Fast	Slow	(Kahneman, 2011)
Unconscious	Conscious	(Wilson, 2002; Uleman, 2004)
Implicit	Explicit	(Reber, 1993)
Low effort	High effort	(Evans, 2008)
High capacity	Limited capacity	(Evans, 2008)
Parallel	Sequential	(Evans, 2008; Lieberman, 2009)
Associative	Rule-based	(Sloman, 1996)
Experiential	Rational	(Epstein, 1994)
Heuristic	Analytic	(Evans, 2006)
Intuitive	Analytic	(Hammond, 1996)
Reflexive	Reflective	(Lieberman et al., 2002)
Holistic	Analytic	(Nisbett et al., 2001)
Nonverbal	Linked to language	(Evans, 2008)
Independent of working memory	Limited by working memory capacity	(Evans, 2009)
Phylogenetically older	Phylogenetically newer	(Lieberman, 2009)

The table summarizes recurring distinctions proposed across different dual-process theories. These characteristics represent broad theoretical tendencies and should not be interpreted as universally shared features of Type 1 and Type 2 processing across all dual-process accounts. A broader comparison of attributes associated with dual-process theories is provided by Evans (2008).

Given its broad applicability, DPT has attracted increasing attention not only in cognitive psychology but also in psychiatry. By distinguishing between different modes of information processing, this framework has been applied to a wide range of domains, from psychopathology, clinical decision-making, and psychotherapeutic change to psychodynamic formulations of the unconscious and neuroscience. Nevertheless, how these different lines of research relate to one another—and whether they offer complementary perspectives on mental functioning—remains a subject of ongoing debate. Accordingly, this review first traces the historical development of DPT and examines its core assumptions and contemporary debates to provide the conceptual background for its current theoretical position. It then considers how DPT may be placed in dialogue with psychodynamic theory, clinical psychiatry, and neuroscience. Rather than translating concepts across these different fields or establishing one-to-one correspondences between their constructs and findings, our aim is to examine whether they offer complementary perspectives on related psychological phenomena while respecting their distinct explanatory levels. Through this integrative approach, we aim to contribute to a more comprehensive understanding of human mental functioning and psychopathology.

To this end, we adopted a narrative, integrative approach. The conceptual framework was developed through theoretical literature, including books and journal articles on DPT and psychodynamic models of mental processing. This theoretical foundation was complemented by searches of PubMed using combinations of terms related to “dual-process theory”, “psychiatry”, “psychopathology”, “neuroscience”, “clinical decision-making”, “psychotherapy”, “psychodynamic”, “psychoanalysis”, and “primary process thinking”. Searches were conducted iteratively as relevant conceptual and clinical themes emerged. Given its conceptual and interdisciplinary scope, no formal inclusion or exclusion criteria or publication-date restrictions were applied. Literature was selected based on its theoretical relevance and contribution to the integrative discussion.

## 2 Historical development of dual-process theories

The idea that the human mind comprises distinct modes of functioning has a long intellectual history, with some of its earliest formulations found in ancient Greek philosophy. In the Phaedrus, Plato portrayed the human soul as involving tensions between reason, passion, and desire, while Aristotle distinguished rational and irrational faculties of the soul. Although these accounts should not be equated with modern DPT, they are widely regarded as early conceptualizations of the idea that the human mind operates through qualitatively distinct modes (Zielonka et al., 2024).

Over the following centuries, philosophers proposed different conceptions of how the human mind operates. In particular, the notion that mental processes can operate outside conscious awareness was developed by Leibniz and Kant during the seventeenth and eighteenth centuries (Thomas, 2009; Leland, 2018), and became more prominent in the nineteenth century through the contributions of Schopenhauer and von Hartmann (Evans and Frankish, 2009). Schopenhauer argued that human behavior—and indeed all existence—is driven by a blind, purposeless, and irrational “will” operating independently of conscious reason. Similarly, Friedrich Nietzsche challenged the notion that behavior could be fully explained by conscious and rational processes, questioning the central role of the conscious self (Weinberger and Stoycheva, 2019). In Philosophy of the Unconscious, von Hartmann provided one of the first systematic philosophical treatments of the unconscious (Evans and Frankish, 2009). Nevertheless, during this period, discussions of the duality of the human mind and the unconscious remained largely philosophical and metaphysical and had not yet been conceptualized as cognitive processing systems in the sense understood by modern psychology.

Beginning in the second half of the nineteenth century, this distinction gradually acquired a more psychological and functional character. Several philosophers and physicians began to conceptualize the unconscious in terms of automatic mental operations underlying everyday cognitive functioning. Early examples include Hermann von Helmholtz's concept of unconscious inferences in perception, Thomas Laycock's account of reflexive brain functions, and William Benjamin Carpenter's notion of unconscious cerebration (Hatfield, 2002; Wilson, 2002). During this period, the unconscious was increasingly viewed as a set of automatic mental processes supporting perception, learning, habits, and behavior. Similarly, although William James remained skeptical of metaphysical interpretations of the unconscious, he emphasized the influence of habits and what he termed “the fringe”—the processes surrounding the focus of consciousness—on thought and behavior (Weinberger and Stoycheva, 2019). Collectively, these developments may be regarded as early antecedents of the cognitive, or adaptive unconscious, that occupies a central place in contemporary DPT.

Another important milestone in the development of modern DPT was the establishment of psychology as an experimental discipline during the second half of the nineteenth century. The psychophysical studies pioneered by Gustav Fechner and Wilhelm Wundt demonstrated that conscious mental processes could be investigated using experimental methods. At the same time, research by the Würzburg School gave rise to debates surrounding “imageless thought”—the idea that certain conscious processes, such as problem solving and judgment, could occur without conscious mental imagery. Furthermore, Francis Galton's word association experiments suggested that a substantial proportion of mental activity operates automatically and outside conscious awareness. Together, these developments brought the distinction between conscious and unconscious mental processes within the scope of experimental psychology (for a comprehensive review, see Evans and Frankish, 2009).

Nevertheless, the rise of behaviorism in the early twentieth century shifted psychology's focus from mental processes to observable behavior. In contrast, Gestalt psychologists challenged the behaviorist view by investigating insight-based problem solving (Evans and Frankish, 2009). They argued that problem solving could not always be explained by the gradual acquisition of habits through trial and error. Instead, they demonstrated that solutions could emerge through the sudden restructuring of one's mental representation of a problem (Vitello and Salvi, 2023). By showing that acquired habits and mental sets can hinder problem solving, they anticipated later distinctions between automatic, habit-based thinking and flexible, reflective forms of cognition. Together with Jean Piaget's theory of cognitive development and Lev Vygotsky's work on inner speech, these developments contributed to growing criticism of behaviorism, culminating in the cognitive revolution of the mid-twentieth century, which restored the study of mental processes to the center of psychology (Evans and Frankish, 2009).

Following the cognitive revolution, processes operating outside conscious awareness increasingly became the subject of experimental investigation. Reber's work on implicit learning was influential, demonstrating that individuals could learn complex patterns and rules without being able to verbalize them (Reber, 1993). In artificial grammar learning experiments, participants classified novel letter strings above chance despite limited explicit knowledge of the grammatical rules. These findings provided evidence for learning outside conscious awareness and contributed to the development of the concept of the cognitive unconscious, establishing one of the key experimental foundations for modern DPT.

The distinction between conscious and unconscious mental processes has occupied a central place not only in cognitive psychology but also in dynamic psychiatry and psychoanalytic theory. In particular, the distinction between primary and secondary process thinking offers a useful framework for dialogue with contemporary DPT, as discussed in the section on the psychodynamic perspective.

## 3 The emergence of modern dual-process theories

Against this historical background, modern DPT began to take shape during the second half of the twentieth century. Models developed within cognitive psychology, decision-making research, and social cognition proposed that the human mind operates through distinct modes of processing, giving rise to the theoretical approaches that collectively constitute DPT.

Research on deductive reasoning played an important role in this development. In particular, studies using the Wason selection task demonstrated that participants' reasoning was heavily influenced by the superficial features of a conditional rule rather than by its underlying logical structure (Wason and Evans, 1974). In this task, participants decide which two of four cards must be turned over to test a conditional rule. Rather than selecting the logically correct cards, most participants choose those explicitly mentioned in the rule. Wason and Evans termed this tendency “matching bias”, arguing that these responses were driven by automatic processes operating outside conscious awareness (Wason and Evans, 1974). Similarly, research on syllogistic reasoning identified “belief bias”, whereby individuals are more likely to accept a logically invalid conclusion if it aligns with their pre-existing beliefs. Conversely, when conclusions conflict with prior beliefs, individuals evaluate them more carefully. Together, these findings suggested that reasoning is shaped not merely by formal logic but by intuitive processes biased by prior knowledge, beliefs, and contextual cues (Evans et al., 1983).

DPT have had one of their greatest impacts in the field of decision-making. Building on their heuristics and biases research program, Daniel Kahneman and Amos Tversky proposed that people frequently rely on fast and intuitive heuristics when making judgments and decisions (Kahneman and Tversky, 1982). They subsequently incorporated these findings into a dual-process framework, associating heuristic judgments that give rise to cognitive biases with System 1, while linking analytic reasoning that monitors and, when necessary, overrides these judgments to System 2 (Kahneman and Frederick, 2002).

Beginning in the 1980s, DPT also became influential in social cognition (Chaiken, 1980; Petty and Cacioppo, 1981). Research in this field has focused on the distinction between automatic, often implicit forms of information processing and more controlled, conscious evaluations that shape phenomena such as person perception, stereotyping, and attitude change (Smith and DeCoster, 2000; Evans, 2008). In addition, research showed that individuals' explicitly stated attitudes and beliefs do not always correspond to implicit attitudes that influence their behavior (Evans, 2008). These distinctions were subsequently reinforced by findings from social cognitive neuroscience, which proposed a reflexive system associated with automatic and associative processing and a reflective system responsible for executive control and conscious evaluation (Lieberman et al., 2002).

Considering this historical development and the accumulated body of research, contemporary DPT may be understood as a family of theories rather than a single unified model, proposing that cognition operates through two distinct modes of processing. Although researchers have described these modes using a variety of terms (Table 1), they are generally characterized by distinctions such as automatic vs. controlled, implicit vs. explicit, intuitive vs. analytic, and associative vs. rule-based processing, with the specific combination and emphasis of these dimensions varying across theoretical accounts. Consequently, the defining characteristics of these processes, the nature of their relationship, and even whether they constitute two distinct and coherent systems remain subjects of ongoing debate.

## 4 Contemporary debates in dual-process theories

Despite their widespread influence across multiple disciplines, DPT remain the subject of considerable conceptual debate. Although broad consensus exists regarding the distinction between two modes of processing, critical questions remain concerning the architecture of these processes, the nature of their interaction, the role of heuristics in human rationality, and whether the characteristics attributed to each process reflect coherent underlying cognitive systems (De Neys, 2017b). Before examining the potential dialogue between DPT and psychodynamic, clinical, and neurobiological perspectives, it is therefore essential to review these ongoing conceptual debates, as they provide the theoretical context for the interdisciplinary discussion that follows.

### 4.1 The architecture and interaction of processes

One of the central debates in the dual-process literature concerns the architecture of Type 1 and Type 2 processing and the nature of their interaction. Several theoretical models have been proposed to explain this relationship. According to the default-interventionist model, Type 1 processes dominate everyday cognition by continuously generating automatic and intuitive responses, whereas Type 2 processing is recruited when necessary to evaluate and, if required, override these responses. In contrast, the parallel-competitive model posits that both processing modes operate concurrently and compete for behavioral control (Lieberman et al., 2002; Evans, 2008). More recently, several authors have argued that these models need not be viewed as mutually exclusive. Hybrid two-stage accounts propose that a monitoring process continuously evaluates potential conflict between Type 1 responses, whereas more effortful Type 2 processing is recruited only when such conflict is detected (De Neys, 2017a).

Beyond differences in the proposed relationship between the two processes, subsequent theoretical developments questioned whether Type 1 and Type 2 processing should be conceptualized as two independent systems at all. Early dual-process models were often interpreted as implying that these two modes constitute separate mechanisms operating side by side. As theoretical discussions evolved, however, this separation came under criticism. In response, Evans (2008) reformulated the distinction by shifting its primary criterion from consciousness to the engagement of working memory. From this perspective, Type 2 processing is conceived as a higher-order hierarchical organization that uses working memory to manipulate, simulate, and transform the autonomous representations generated by Type 1 processes. This perspective may thus be conceived as shifting the relationship between the two processes from a horizontal to a vertical, hierarchical conceptualization.

Several contemporary theories of mind have likewise proposed that conscious and reflective processing emerges as parallel and automatic biological processes become serial, organized, and rule-governed through language (Dennett, 1991). Accordingly, the biological brain consists of specialized subsystems that operate in parallel and compete for behavioral control, whereas the conscious mind is a kind of “virtual organization” that emerges through learned behaviors such as language and, particularly, inner speech. Reflective processing can therefore be understood as a higher-order, serial mode of organization built upon these parallel biological processes and responsible for regulating their activity.

Related accounts have suggested that conscious, rule-based, and reflective processing constitutes a higher-order organization built upon parallel, associative, and often unconscious processes (Smolensky, 1988; Frankish, 2004; Carruthers, 2009). In this view, Type 2 processing is conceptualized not as a separate rational module, but as the recursive and conscious use of Type 1 mechanisms (Carruthers, 2009). Reflective processing is thought to emerge through the mental rehearsal of actions and evaluation of their cognitive and emotional consequences. Collectively, these perspectives suggest that the processes described within DPT may be better understood not as two independent systems operating side by side, but as a hierarchical organization in which Type 2 processing emerges from the higher-order organization of Type 1 processes.

### 4.2 Rationality and heuristics

Another important debate within DPT concerns rationality. Early DPT largely viewed fast and intuitive processes as the primary source of cognitive biases (Kahneman and Frederick, 2002). This view was later challenged by several researchers (Evans and Over, 1996; Gigerenzer et al., 1999). It was argued that failure to conform to the normative rules of logic or probability theory should not automatically be regarded as irrational, and that many forms of thinking regarded as errors in experimental studies may be functional and adaptive in everyday life (Evans and Over, 1996). Similarly, Gigerenzer (2007) argued that the superiority of analytical thinking has been demonstrated mainly in environments with clear and well-defined rules, such as laboratory settings, whereas in real-world environments characterized by uncertainty, limited information, and time pressure, intuitive heuristics can lead to more effective decisions. This view is captured by the concept of “ecological rationality”, according to which the rationality of a decision depends not on its conformity to abstract normative rules, but on how well it fits the structure of the environment and serves the individual's goal (Gigerenzer, 2025).

Stanovich and West (2003) offered a more integrative perspective by acknowledging both the adaptive value and limitations of intuitive processing. They argued that cognitive tendencies that are evolutionarily adaptive do not always produce functional outcomes in modern technological societies. Thus, although Type 1 processing is effective and adaptive in many situations, it may give rise to systematic cognitive biases, particularly when abstract, statistical, or decontextualized reasoning is required. Stanovich and West (2003) further emphasized that humans are not limited to relying on evolutionarily shaped intuitions, but possess the cognitive capacity to evaluate and, when necessary, override these tendencies and act in accordance with their individual goals.

More recent developments have refined this debate by emphasizing that rationality is a property of the cognitive agent as a whole rather than of the cognitive mechanisms that generate responses (Evans and Stanovich, 2013). Therefore, Type 1 and Type 2 processes are neither rational nor irrational but simply more or less efficient. Consequently, their contribution to global rationality depends on how these mechanisms are utilized by the agent. This framework helps explain why Type 1 processing may support expert intuition, while Type 2 processing, despite its analytical capacity, may sometimes serve to rationalize pre-existing beliefs or motivations rather than correct them (De Neys, 2017a; Melnikoff and Bargh, 2018). Together, these findings suggest that the relationship between DPT and rationality is considerably more nuanced than originally proposed, with both Type 1 and Type 2 processing capable of contributing to either adaptive or biased reasoning depending on the task, context, and individual goals.

### 4.3 Conceptual critiques and contemporary developments

Beyond debates concerning architecture and rationality, DPT have been the subject of broader conceptual critiques regarding their theoretical foundations (Frankish, 2009; Melnikoff and Bargh, 2018). It has been argued that dividing the mind into two distinct systems may represent an oversimplified metaphor rather than an accurate account of cognitive architecture. Moreover, constructs such as Type 1 and Type 2 processing have been criticized as merely redescribing the cognitive phenomena they are intended to explain. According to this view, unless the mechanisms underlying these systems are independently specified, DPT risks committing the nominal fallacy (Melnikoff and Bargh, 2018).

A related criticism concerns the assumption that the characteristics attributed to Type 1 and Type 2 processing necessarily co-occur. For example, automaticity, unconscious processing, speed, and low cognitive effort are often treated as inseparable properties of a single processing mode, yet empirical findings suggest that these characteristics do not always cluster together (Frankish, 2009; Melnikoff and Bargh, 2018). This issue has been termed the “alignment problem,” and DPT has been criticized for failing to adequately explain why these features tend to cluster in particular ways (Melnikoff and Bargh, 2018).

Related to this critique, Bellini-Leite (2022) addressed the unity problem, which asks why the characteristics attributed to each type of processing tend to occur together as coherent clusters. To account for this, Bellini-Leite (2022) proposed that these processes are grounded in distinct cognitive architectures, with the unity of Type 1 processing arising from embodied predictive processing and that of Type 2 processing from symbolic classical cognition. Within this framework, Type 1 processing is associated with predictive mechanisms that continuously generate and update expectations through the organism's interactions with its environment, whereas Type 2 processing depends on a more reflective cognitive architecture operating over symbols, rules, and working memory (Bellini-Leite, 2022).

Taken together, these debates suggest that our understanding of DPT—and, more broadly, of the architecture of the human mind—continues to evolve. Against this background, it may be useful to consider whether related questions have been addressed within other theoretical traditions. Long before such issues became central to contemporary cognitive psychology, psychodynamic theory had already proposed that mental functioning is organized across multiple modes of processing. Although developed from a different theoretical perspective, these ideas offer a valuable framework for placing contemporary DPT in dialogue with psychodynamic concepts.

## 5 Psychodynamic perspective

Psychodynamic theories and DPT emerged from different theoretical traditions, yet both seek to explain the organization of mental functioning. A central premise of psychodynamic theory is that a substantial portion of mental life operates outside conscious awareness. Psychodynamic theory developed a rich clinical account of the unconscious and the principles underlying its organization, addressing many questions that remain central to cognitive science today. Revisiting these ideas offers a valuable opportunity to reconsider contemporary questions in cognitive science from a different perspective. Accordingly, the following sections review psychodynamic conceptions of the unconscious, trace the development of primary process thinking from Freud to modern psychoanalytic models, and examine the conceptual relationships between these concepts and DPT.

### 5.1 The dynamic unconscious

Toward the end of the nineteenth century, the concept of the unconscious became central to psychiatry and psychopathology. During this period, the work of Pierre Janet and the psychoanalytic theory developed by Sigmund Freud proposed that a substantial part of human behavior is shaped by mental processes operating outside conscious awareness (Weinberger and Stoycheva, 2019). Janet introduced the concept of “psychological automatism” to describe mental processes that operate automatically outside voluntary control, proposing that the mind is composed of numerous automatic processes (Ellenberger, 1970). According to Janet, consciousness normally integrates these processes into a coherent personality; however, trauma or overwhelming stress may lead to their dissociation, allowing them to continue operating outside awareness and contributing to psychopathological symptoms (Weinberger and Stoycheva, 2019). In doing so, Janet provided one of the first systematic psychological accounts of mental processes operating outside conscious awareness.

Freud subsequently placed the unconscious at the center of psychological theory, arguing that psychological processes cannot be fully understood without reference to unconscious mental life. In his topographical model, the mind was organized into the conscious, preconscious, and unconscious systems (Freud, 1957, 1965). Whereas the conscious and preconscious systems were organized rationally and linguistically, the unconscious was governed by drives and operated according to qualitatively different principles (Weinberger and Stoycheva, 2019). Freud described the characteristic mode of functioning of the unconscious as primary process thinking, whereas the rational, reality-oriented mode of the conscious and preconscious systems was termed secondary process thinking.

Freud later recognized that levels of consciousness did not correspond directly to mental structures. Accordingly, in his structural model, he reconceptualized the mind as the id, ego, and superego, and proposed that unconscious processes could be found in all structures (Freud, 1989). Following Freud, ego psychology, object relations theory, and other psychodynamic approaches expanded the conception of the unconscious. While ego psychology emphasized the adaptive and organizational functions of the ego, many of which operate outside conscious awareness, object relations theory placed early relational experiences at the center of unconscious processes. Consequently, the unconscious was increasingly conceptualized not merely as a reservoir of repressed drives, but as a broader domain of mental functioning responsible for organizing human experience (Rapaport, 1960; Weinberger and Stoycheva, 2019).

With these theoretical developments, the conceptualization of primary and secondary processes also evolved. They no longer strictly mapped onto separate conscious and unconscious systems. Instead, mental functioning came to be viewed as existing along a continuum, with mental operations displaying primary and secondary process characteristics to varying degrees (Arlow and Brenner, 1964). These developments will be revisited in subsequent sections, where Freud's original distinction between primary and secondary process thinking, its later reformulations within psychodynamic theory, and its relationship to contemporary DPT are discussed.

### 5.2 Psychoanalytic dual-process model: primary and secondary process thinking

The unconscious has remained a cornerstone of psychoanalytic theory, yet the primary process—Freud's original account of how the unconscious operates—has largely remained in the background (Robbins, 2018). This may partly reflect Freud's inconsistent use of the concept. Another reason may be that, in an effort to develop more empirically grounded theories, subsequent psychoanalytic approaches often described related forms of mental functioning using alternative concepts and terminology rather than referring to Freud's primary process (Robbins, 2018). Nevertheless, because it proposes a fundamental distinction between two modes of mental functioning—one that invites dialogue with contemporary DPT—and offers a valuable framework for understanding the organization and functioning of the mind, the concept of primary process thinking warrants further consideration.

In its classical definition, primary process thinking refers to unconscious mental activity in which psychic energy flows freely and uninhibitedly from one idea to another (Freud, 1957). Freud first developed the concept in his unpublished 1895 manuscript “Project for a Scientific Psychology” and later introduced it into the psychoanalytic literature through “The Interpretation of Dreams” (Freud, 1965). Freud referred to this mode of mental functioning as “primary” because he regarded it as emerging earlier in both ontogeny and phylogeny.

Freud's distinction between primary and secondary process thinking is based on differences in the mobility of psychic energy (cathexis). In primary process thinking, psychic energy remains relatively mobile, and mental representations are organized around affective and instinctual associations rather than logical relations. Importantly, Freud did not regard primary process thinking as random or chaotic, but as a distinct mode of mental organization governed by principles different from those of secondary process thinking. Within this mode, representations serving similar emotional functions may become interchangeable; the boundaries between subject and object become blurred; a part may stand for the whole (pars pro toto); and thoughts may be experienced as exerting a direct influence on reality. The characteristic mechanisms of primary process thinking—including displacement, condensation, perceptual identity, and tolerance of contradiction—constitute the basis of dream formation (Freud, 1965). Freud further proposed that the mind possesses a compulsion to associate, continually forming connections based on superficial similarities and shared features rather than logical relationships (Freud, 1960, 1965). Overall, primary process thinking is characterized by permeable category boundaries, the predominance of associative and analogical links over logical consistency, and the ready formation of metaphorical connections.

In contrast, according to Freud, secondary process thinking is characterized by more stable and controlled cathexes. Mental representations are organized according to logical and experience-based relationships, and mental activity is guided by the reality principle (Holt, 2008). Consequently, thinking becomes increasingly reality-oriented and goal-directed, enabling planning, anticipation of consequences, evaluation of alternatives, and behavioral regulation.

Freud regarded dreams and the irrational features of psychotic symptoms as prototypical manifestations of primary process thinking. Fantasies and magical thinking were likewise considered phenomena that reflect the characteristics of primary process thinking (Robbins, 2018). However, this classical formulation was reinterpreted by later psychoanalytic theorists.

### 5.3 Reconceptualizing the functioning of the unconscious and primary process

Subsequent psychoanalytic theorists substantially broadened Freud's conception of primary process thinking. One of the earliest developments emerged from observations of hypnagogic states, which suggested that as the level of consciousness decreases, thinking becomes more imagistic and associative (Rapaport, 1951). Within this framework, every thought was proposed to undergo a process of “microgenesis” beginning with the associative organization characteristic of primary process and gradually developing into the logical structure of secondary process (Rapaport, 1951). These developments marked the beginning of a broader reconceptualization of primary process. Rather than viewing it as confined to unconscious functioning, dreaming and psychopathology, subsequent theorists increasingly conceptualized it as a more general mode of mental organization that operates in everyday mental life, including creativity, imagination, and artistic production. From this perspective, primary and secondary processes are better viewed as complementary modes of mental organization that can occur to varying degrees under different conditions rather than as direct equivalents of the conscious and the unconscious (Bazan, 2024).

One of the most systematic attempts to explain mental organization in terms of general principles was proposed by Matte Blanco (1975). Matte Blanco conceptualized mental functioning as a layered structure extending along a continuum from consciousness to the unconscious and explained this organization in terms of two fundamental modes: asymmetrical and symmetrical logic. The continual coexistence of these two modes was termed “bi-logic” (Matte Blanco, 1975). At the upper levels of this layered organization, asymmetrical logic predominates and supports rational, reflective, and symbolic thought by differentiating objects and preserving temporal and causal relationships. At deeper levels, symmetrical logic becomes increasingly dominant and resembles the characteristics Freud attributed to primary process thinking. Within this mode, distinctions between objects become blurred, temporal and causal relations weaken, contradictions can coexist, and similarity is experienced as identity (Badalamenti, 2018). Consequently, mechanisms such as displacement and condensation can be understood as expressions of symmetrical logic. Similarly to Matte Blanco, Badalamenti (2018) argued that primary process thinking is not random but follows the principle of equivalence. Mental representations become associated because they partially satisfy the same wish or emotional function, thereby forming associative networks based on shared features (Badalamenti, 2018). Moreover, the first emotionally significant representative of a class comes to define the class as a whole, with subsequent members being perceived through the emotional meaning established by that representative. As Matte Blanco (1975) stated, the unconscious “knows classes, not individuals.” In this way, Matte Blanco reformulated Freud's concept of primary process as a more general principle of mental organization. This distinction also closely parallels several dimensions of DPT, including associative vs. rule-based processing, parallel vs. sequential organization, and holistic vs. analytic thinking.

Other psychoanalytic traditions likewise described modes of mental organization that share characteristics with Freud's notion of primary process, although they did so using different conceptual frameworks and terminology. Within object relations theory, one example is the concept of unconscious “phantasy” (Robbins, 2018), which Isaacs (1948) described as a pre-reflective mode of organization characterized by sensory, perceptual, bodily, and motor experience. At this level, experience has not yet acquired symbolic or reflective representation, and the boundaries between subject and object remain poorly differentiated. Likewise, the absence of temporal organization, contradiction, and negation parallels the characteristics of Freud's conception of primary process thinking.

A similar development emerged within relational and attachment-oriented psychoanalytic theories, which shifted the emphasis from repressed contents to forms of experience that have not yet acquired symbolic representation. Within this framework, the unconscious was reconceptualized as a level of mental organization in which sensory, bodily, affective, and interpersonal experiences are organized without being translated into words or becoming the object of conscious reflection. In this view, the unconscious consists of “what the body knows but the mind has not yet grasped or articulated” (Robbins, 2018). This form of unconscious functioning has been described in terms of implicit knowledge or unformulated experience (Stern, 1997). More specifically, implicit knowledge has been conceptualized as a pre-symbolic mode of mental representation that emerges through the moment-to-moment rhythms, gestures, and silences shared between the infant and caregiver (Boston Change Process Study Group, 2007). Together, these perspectives reconceptualize the unconscious not merely as a repository of experiences lacking symbolic representation, but as a distinct level of mental organization in which sensory, bodily, affective, and interpersonal experiences are organized according to their own principles.

Among these formulations, Wilma Bucci's Multiple Code Theory provides one of the most systematic accounts of the mental organization of pre-symbolic and symbolic experiences. By reformulating the psychoanalytic concept of primary process within contemporary cognitive models of information processing, Bucci (1997, 2011) proposed a hierarchical system consisting of three modes of processing: subsymbolic nonverbal, nonverbal symbolic, and verbal symbolic. The subsymbolic nonverbal system encompasses bodily functioning, procedural and implicit memory, as well as the physiological aspects of emotion. Contrary to common assumptions, it is not merely a collection of chaotic bodily sensations; rather, it constitutes an organized form of unconscious information processing that remains non-symbolic because it contains neither words nor images. Bucci (1997) argued that these subsymbolic processes, characterized by sensory, perceptual, bodily, and motor features, form organized systems whose complexity and scope continue to develop throughout the lifespan. Examples include the rapid evaluation of facial expressions, detecting threat from body posture, and perceiving emotional nuances in tone of voice. Bucci suggested that this mode of processing is experienced in everyday life as intuition and that emotional communication between people occurs primarily at this subsymbolic level. In this regard, she argued that Reik's (1948) concept of “listening with the third ear” is fundamentally grounded in this form of subsymbolic communication (Solano, 2010).

In contrast, Bucci's symbolic systems represent the levels at which experience is organized through images and words. The nonverbal symbolic system transforms emotional experience into visual imagery and dream thought, thereby giving it mental representation, whereas the verbal symbolic system translates these representations into language, enabling reflection on one's experience as well as the recognition and regulation of emotions. Under healthy conditions, these three systems remain interconnected, allowing bodily experiences originating at the subsymbolic level to become progressively represented in mental imagery and, ultimately, in words (Bucci, 2002, 2011).

Building on these developments, Robbins (2018) proposed an even broader reformulation of primary process. He argued that primary process thinking constitutes a fundamental principle of organization not only for the unconscious but also for certain forms of consciousness (Robbins, 2018). To integrate related phenomena that had been described using different concepts, he introduced the concept of “primordial consciousness”, a mode of awareness observed in both normal and pathological states that is conscious, but not reflective, and in which cognitive, affective, sensory, perceptual, and motor processes remain closely intertwined. Robbins conceptualized this mode as the earliest level of mental organization preceding the development of reflective functioning—a body-mind in which bodily sensations, emotions, perception, and action have not yet become differentiated. At this level, concrete images and sensations predominate over representations and symbols. He emphasized that this primordial mode of organization is not confined to psychopathology but can also be observed in parent–infant interactions, religious or spiritual experiences, creative experiences, and contexts of collective emotional experience.

Taken together, these psychoanalytic perspectives suggest that primary process has increasingly been conceptualized as a mode of organization characterized by generalizing and associative processes, as well as sensory, bodily, affective, and pre-reflective forms of experience. Although these theories employ different terminology, they converge in proposing the existence of an organized and functional level of mental organization that precedes reflective thought. Collectively, these developments provide an important conceptual bridge between psychoanalytic theories and contemporary dual-process accounts.

### 5.4 Dialogue between psychoanalytic theories and dual-process theories

Psychodynamic theories and contemporary DPT are two distinct theoretical traditions that explain mental processes at different levels of analysis. Rather than treating their concepts as direct equivalents, a dialogue between these traditions seeks to examine how similar mental phenomena have been conceptualized within different theoretical frameworks. Such a dialogue may facilitate the re-examination of psychodynamic concepts such as the unconscious and primary process thinking using experimental cognitive methods while contributing to a more comprehensive understanding of the experiential, motivational, developmental, and interpersonal aspects of the distinctions proposed by DPT.

Attempts to establish such a dialogue have been made for decades. Dixon (1956) reported that words presented above the threshold of awareness elicited direct and logical associations, whereas the same stimuli presented subliminally gave rise to more metaphorical associations. Similarly, Spence and Holland (1962) suggested that conscious awareness constrains the range of associations activated by a stimulus, whereas unconscious processing permits broader and more diverse associative activation. These findings raised the possibility that unconscious processes may be organized according to associative principles resembling those Freud described as primary process thinking.

Subsequently, several authors emphasized the motivational aspects of unconscious processing and suggested that some fundamental human motives are not always accessible to conscious awareness (Atkinson and McClelland, 1948; Shevrin and Dickman, 1980). Subsequent research on implicit motives supported this view, showing that implicit and explicit motivational systems operate partly independently and may influence behavior through different pathways (Schultheiss et al., 2008).

Other researchers have sought to investigate the characteristics of primary process thinking more directly through experimental methods (Brakel, 2004; Bazan and Brakel, 2023). To this end, they related primary and secondary process thinking to two forms of categorization described in cognitive psychology. Of these, attributional similarity refers to grouping objects based on shared perceptual or surface features, whereas relational similarity involves categorization according to the relationships and conceptual connections between objects (Medin et al., 1990). For example, while classifying whales together with fish reflects attributional similarity, classifying them as mammals reflects relational similarity (Arminjon, 2011). Brakel and colleagues proposed that these two forms of cognitive organization parallel primary and secondary process thinking, respectively (Brakel, 2004). Using the Geometric Categorization Task (GeoCat), in which participants classify geometric figures according to either perceptual similarity or relational structure, they found that subliminal presentation promoted categorization based on attributional similarity, whereas supraliminal presentation favored relational similarity (Brakel, 2004). They reported that attributional patterns were more pronounced in children, consistent with the view that primary process-like modes of organization emerge earlier in development. Similar patterns were observed in individuals with higher levels of anxiety and with psychosis (Brakel and Shevrin, 2005; Bazan et al., 2013). In subsequent work, Cutler and Brakel (2014) argued that primary process should be understood not merely as an associative cognitive mechanism but also as a mode of mental organization grounded in affective and bodily processes. This perspective is broadly consistent with embodied approaches in cognitive science (Bellini-Leite, 2022).

Taken together, these experimental approaches suggest that specific characteristics associated with primary process thinking can be investigated using methods derived from cognitive psychology. However, how primary process thinking can be related to contemporary DPT depends partly on how the concept itself is theoretically defined. In this regard, it may be useful to distinguish between approaches that conceptualize primary process as a qualitatively distinct mode of mental organization and those that view it as reflecting a more graded form of differentiation. Authors such as Robbins and Bucci conceptualize primary process as a pre-verbal and non-reflective mode of representation and experience that operates primarily through sensory, affective, bodily, and motor forms of experience (Bucci, 2011; Robbins, 2018). Secondary process, by contrast, is characterized by more linguistic, reflective, sequential, and logical modes of organization. Within these perspectives, primary and secondary processes are therefore understood as qualitatively distinct modes of mental organization.

In contrast, authors such as Badalamenti and Matte Blanco explain primary process and unconscious functioning in terms of organizational principles that resemble those underlying conscious cognition, emphasizing differences of degree rather than kind (Matte Blanco, 1975; Badalamenti, 2018). In Matte Blanco's formulation, although mental functioning is described in terms of symmetrical and asymmetrical logic, these do not constitute two entirely separate logical systems (Badalamenti, 2018). Rather, his stratified model conceptualizes mental functioning as a continuum in which the relative proportions of symmetry and asymmetry gradually change across different levels of consciousness. Similarly, Badalamenti (2018) argues that secondary process can be understood as a more differentiated form of primary process, in which objects are progressively distinguished based on an increasing number of attributes and categories become increasingly specific. Whereas primary process operates with broader classes, secondary process functions through narrower and more highly differentiated categories.

These differences suggest that the dialogue between psychoanalytic primary process thinking and contemporary DPT may not be unitary. Depending on whether primary process is conceptualized as a qualitatively distinct representational mode (e.g., Robbins and Bucci) or as a less differentiated form of the same cognitive organization (e.g., Matte Blanco and Badalamenti), different points of convergence with DPT may emerge. The emphasis of Matte Blanco and Badalamenti on associative organization appears particularly compatible with DPT accounts that distinguish between associative and rule-based modes of processing. By contrast, Robbins' and Bucci's emphasis on pre-symbolic, sensory, affective, bodily and motor aspects of mental functioning may be more closely related to embodied cognition approaches, which conceptualize Type 1 processing as grounded in bodily experience rather than symbolic representation (Figure 1).

**Figure 1:**
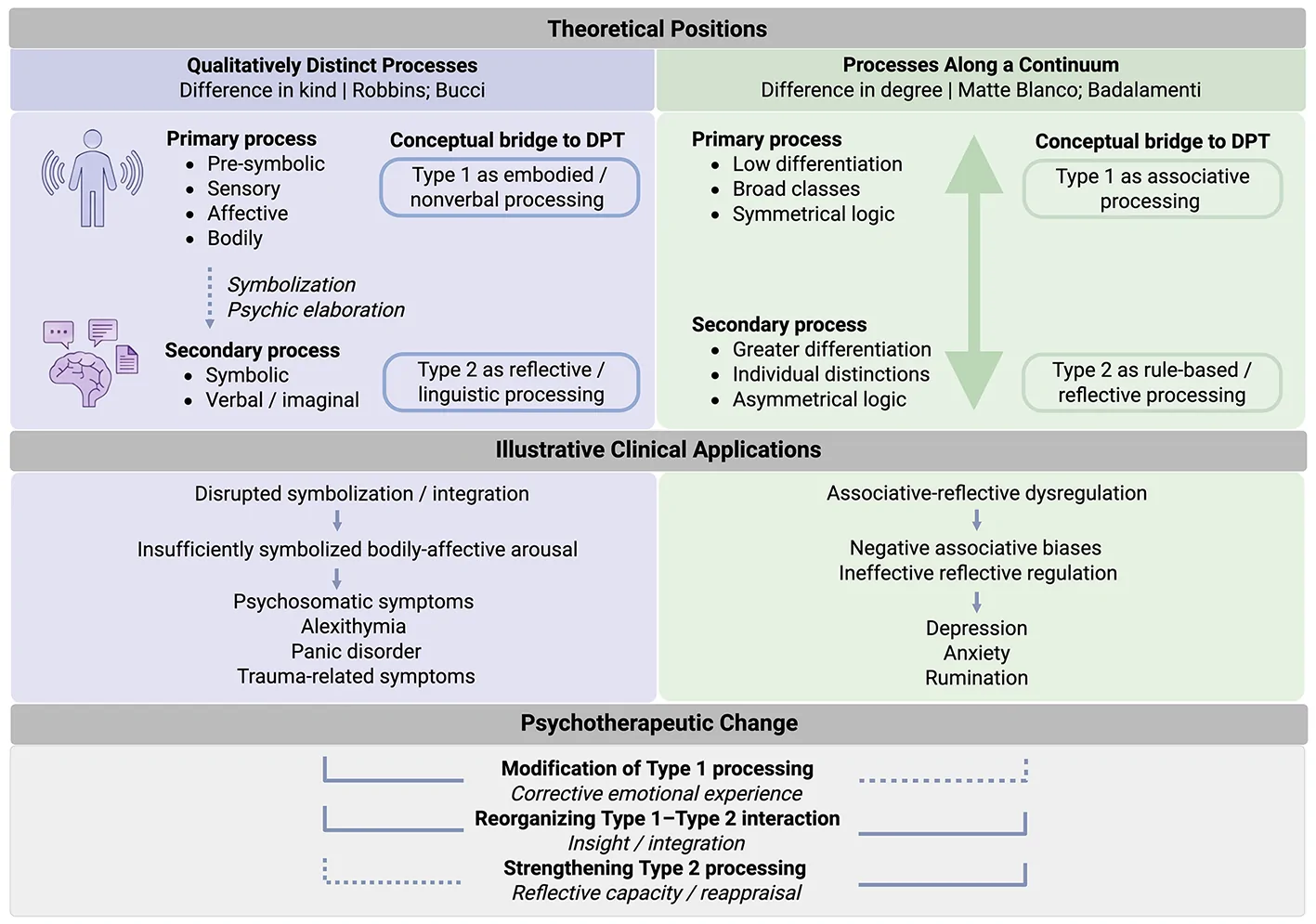
Alternative conceptualizations of primary and secondary process thinking: bridges to dual-process theories and clinical applications. Different conceptualizations of primary-secondary process thinking provide distinct bridges to DPT, highlighting different clinically relevant mechanisms. The correspondences between primary-secondary process models and DPT represent conceptual parallels rather than direct equivalences. The clinical phenomena shown are illustrative examples of these mechanisms and should not be interpreted as uniquely associated with either conceptualization. Similarly, the proposed routes to psychotherapeutic change are non-exclusive and not restricted to either theoretical position. In the therapeutic change section, solid lines indicate greater conceptual emphasis, whereas dashed lines indicate less emphasized but compatible routes.

Accordingly, a productive dialogue between psychodynamic theories and DPT may depend less on treating concepts as direct equivalents than on examining how different forms of mental representation, organizational principles, and processing characteristics have been conceptualized across these traditions. These conceptual distinctions may have important methodological implications, as whether primary and secondary processes are understood as qualitatively distinct modes of organization or as differing degrees of differentiation may influence how these constructs are operationalized and the experimental methods used to investigate them. Such an approach may contribute to a more comprehensive understanding of the complexity of human mental functioning while guiding future research toward a more precise integration of psychodynamic and cognitive models.

## 6 Clinical perspective

Beyond their contribution to understanding mental functioning, DPT may also provide a useful conceptual framework for examining clinical issues such as psychopathology, clinical decision making, and psychotherapeutic change. Accordingly, researchers from diverse theoretical traditions have attempted to explain psychiatric symptoms, clinicians' decision-making processes, and mechanisms of therapeutic change in terms of different modes of mental processing.

### 6.1 Dual-process theories in understanding psychopathology

Applications of DPT to psychopathology can take different forms. Some approaches focus on the relationship between bodily and emotional experiences and their symbolic processing, whereas others emphasize the interaction between fast, automatic processes and slower, more reflective modes of processing. Although these perspectives are not mutually exclusive, they have generally been applied to different clinical phenomena. The former may be particularly useful for understanding alexithymia, somatization, and trauma-related symptoms, whereas the latter has been more widely applied to conditions such as depression and anxiety (Figure 1). Therefore, rather than providing a single explanatory model of psychopathology, DPT may be viewed as a flexible conceptual framework for discussing various clinical phenomena in terms of different mental processes.

#### 6.1.1 Psychosomatic symptoms, somatization, and alexithymia

Several theoretical approaches have been proposed to explain psychosomatic symptoms. Among these, Bucci's Multiple Code Theory provides a useful framework by emphasizing the relationship between bodily and emotional experience and symbolic processing (Bucci, 1997). According to this theory, healthy mental functioning depends on the integration of the subsymbolic nonverbal, nonverbal symbolic, and verbal symbolic systems (Bucci, 2011). Through this integration, bodily and emotional experiences can be processed and symbolized through images and words. Disruptions in the connections among these systems may contribute to psychosomatic symptoms. When traumatic experiences or intense emotional arousal cannot be adequately linked to the symbolic systems, emotional experience remains as “nameless” subsymbolic arousal that cannot be transformed into words or images. This subsymbolic arousal may also be related to chronic stress activation in clinical medicine (Solano, 2010). In such cases, rather than being processed psychologically, experience tends to be expressed through bodily systems, contributing to functional disorders (e.g., irritable bowel syndrome and functional cardiac symptoms) or to structural diseases in which chronic stress contributes to the damage to target organs (Solano, 2010).

This framework also provides a useful basis for understanding alexithymia, a construct closely associated with psychosomatic disorders (Taylor, 2010). Nemiah and Sifneos, who introduced the concept, distinguished between emotion and feeling. They used the term emotion to refer to the biological and physiological components of emotional arousal, whereas feeling denoted the conscious subjective experience of that arousal (Nemiah, 1977). Similarly, researchers such as LeDoux and Panksepp have argued that emotions arise within bodily and neurobiological systems operating outside awareness, whereas conscious feeling emerges through the symbolic representation of these underlying processes (LeDoux, 1996). In Bucci's model, emotion schemas are likewise conceptualized as multilayered structures that encompass sensory, visceral, and motor processes, together with their imaginal and verbal representations. The ability to become aware of one's feelings and link them with thoughts, fantasies, and memories facilitates the regulation of emotional arousal elicited by stressful experiences (Taylor, 2010). Nemiah referred to the process through which somatic arousal is transformed into symbolic forms as “psychic elaboration”. This process involves differentiating and labeling emotions, generating images and fantasies associated with them, and activating related memories and associations. When this mechanism is disrupted, individuals may experience intense emotional arousal yet remain unable to identify or label their feelings or distinguish among different emotions. From this perspective, alexithymia can be understood as a disruption in the integration of the physiological and symbolic dimensions of emotional experience.

The developmental origins of these processes have been conceptualized within attachment theory. Secure attachment relationships provide the interpersonal context in which a child's emotional experiences can be recognized, labeled, and made meaningful by the caregiver. Through these interactions, symbolic capacity and reflective functioning gradually develop, enabling bodily and emotional experiences to be processed through images and words (Fonagy and Target, 1997; Meins et al., 1998). In contrast, childhood neglect and traumatic experiences may disrupt this developmental process and weaken the connections among the subsymbolic, imaginal, and verbal components of emotional experience. In the absence of a secure attachment figure, traumatic experiences cannot be adequately symbolized and remain insufficiently processed within implicit memory networks. Although such dissociations may initially serve a defensive or protective function, over time they may contribute to difficulties in emotion regulation, alexithymia, somatic symptoms, and psychosomatic disorders. Clinical observations and empirical studies suggest that individuals with greater capacities for symbolization, mentalization, and emotion regulation show greater psychological resilience to trauma and everyday stressors (Taylor, 2010).

Dissociation can likewise be understood in terms of disruptions in the integration of these systems. Authors drawing on the tradition of Pierre Janet have argued that the primary mechanism underlying somatoform disorders is dissociation rather than repression (Taylor, 2010). From this perspective, the central problem lies in the failure to adequately integrate experience, resulting in disrupted connections among mental systems. Somatization is thus understood as an excessive preoccupation with bodily sensations arising from subsymbolic experience that remains disconnected from symbolic processing, whereas dissociative symptoms are thought to emerge when different components of emotional schemas become segregated from one another. Consistent with this view, empirical studies have shown that dissociative symptoms frequently co-occur with somatization and that this association is particularly common among traumatized individuals with difficulties in emotion regulation (Van der Kolk et al., 1996).

Taken together, these models of psychosomatic disorders, somatization, alexithymia, trauma-related symptoms, and dissociative phenomena share the view that psychopathology is better understood not primarily in terms of the repression of specific drives, but as clinical manifestations of disrupted integration between implicit, bodily, and affective modes of processing and symbolic, verbal, and reflective modes of processing. In this respect, these models highlight the clinical significance of how implicit and body-based processing is balanced and integrated with reflective and symbolic processing, a perspective that closely parallels themes emphasized in contemporary DPT.

#### 6.1.2 Anxiety disorders

Anxiety disorders can be approached within DPT from two complementary perspectives that differ in the aspects of mental functioning they emphasize. The first, similar to accounts of psychosomatic disorders, focuses on the transformation of bodily and emotional experiences into symbolic forms. The second is associated with Beck's cognitive model and emphasizes the interaction between fast, automatic information processing and slower, more controlled evaluative processes.

One clinical manifestation of impaired symbolic processing and emotion regulation may be panic disorder (Taylor, 2010). According to Nemiah, panic attacks arise when intense emotional arousal cannot adequately engage in the mental process that he termed “psychic elaboration”. Although the individual experiences intense physiological activation, the symbolic means necessary to process this experience are insufficient, and emotional arousal is expressed primarily through bodily symptoms. For this reason, Nemiah described panic disorder as the “prototypical psychosomatic disorder” (Nemiah, 1984).

A second line of explanation relates anxiety disorders to cognitive models that emphasize the interaction between automatic processing and reflective control. This perspective will be discussed in greater detail in the following section on depression.

#### 6.1.3 Depression

Cognitive models of depression have long proposed that negative schemas concerning the self, the world, and the future give rise to systematic biases in information processing and play a central role in the development and maintenance of depressive symptoms (Beck, 1979). Dual-process theories provide a complementary framework for conceptualizing the cognitive mechanisms involved in depression. According to this perspective, mental functioning emerges from the interaction between fast, automatic, and largely associative Type 1 processes and slower, rule-based, and reflective Type 2 processes. Depression is thought to arise and persist because of disruptions in the balance between these two modes of processing (Beevers, 2005).

According to dual-process models, vulnerability to depression is associated with strong associative links between the self and negative contents. The fast associative system interprets new experiences through previously learned associations. These associations are often not consciously accessible, yet they can be readily activated by negative mood states or stressful life events. Consequently, new situations may be interpreted in terms of failure, worthlessness, or inadequacy before conscious evaluation. Consistent with this view, studies have shown that depressed individuals exhibit stronger associative links between the self and negative attributes than healthy controls (Dozois and Dobson, 2001).

The development of depressive symptoms is determined not only by the content of the associative Type 1 processes but also by the extent to which reflective Type 2 processes can modify their outputs. Under healthy conditions, the reflective system can monitor, reinterpret, and, when necessary, correct the initial appraisals generated by associative processing. In individuals vulnerable to depression, however, this corrective function may fail either because the reflective system is not successfully engaged or because, despite being engaged, it is unable to effectively modify the outputs generated by associative processing (Beevers, 2005).

Failure to engage the reflective system may result from limitations in cognitive resources. Life stress, cognitive load, fatigue, and dysphoric mood may reduce working memory capacity, thereby diminishing the effectiveness of reflective processing. Under such conditions, individuals may lack sufficient cognitive resources to evaluate automatically activated negative associations, allowing the outputs of associative processing to dominate the stream of thought. Consistent with this account, studies have shown that individuals with remitted depression interpret ambiguous stimuli more negatively than healthy controls under cognitive load (Wenzlaff and Bates, 1998). Similarly, life stress has been shown to reduce working memory capacity and to contribute to the increased accessibility of depressive thoughts (Klein and Boals, 2001). In addition, reflective processing capacity may itself be limited. Studies have shown that depressed individuals are less likely to engage in reflective processes that reappraise negative self-evaluations (Sheppard and Teasdale, 2000). Moreover, low levels of metacognitive abilities have been associated with an increased risk of depressive relapse (Teasdale et al., 2002).

In the second scenario, even when the reflective system is successfully engaged, it may fail to effectively modify the negative outputs generated by associative processing. As a result, rather than correcting associative biases, reflective processing may instead contribute to their maintenance. Rumination represents one of the clearest examples of this process. It is defined as repetitive and unproductive thinking focused on one's negative mood, experiences, and self-related concerns. Although rumination appears to involve reflective processing, from a dual-process perspective it often serves to reinforce rather than correct negative associative patterns (Beevers, 2005). Thus, in depression, the problem lies not only in the presence of automatic negative thoughts but also in the failure of Type 2 strategies intended to regulate them.

Within dual-process models, emotion regulation also contributes to the mechanisms that maintain depression. Negative themes activated by the associative system intensify dysphoric mood, while the resulting negative affect, in turn, facilitates the activation of further negative associations. Consequently, a reciprocal feedback loop develops between cognitive content and mood. According to Forgas, under normal circumstances, once negative mood reaches a certain intensity, it prompts individuals to engage more reflective (Type 2) strategies aimed at regulating their emotional state (Forgas, 2000). In depression, however, this regulatory mechanism fails, giving rise to a cycle in which negative thoughts and dysphoric mood continuously reinforce one another. Moreover, intrusive thoughts and persistent mental preoccupation associated with negative mood further deplete the cognitive resources required for Type 2 processing, making it increasingly difficult to interrupt this cycle. Taken together, these findings suggest that depression is maintained through disturbances in both associative Type 1 and reflective Type 2 processing, as well as in the dynamic interaction between these two processes.

### 6.2 Dual-process theories in clinical decision-making

Clinical decision-making is a complex process that involves evaluating the patient's multidimensional symptoms, integrating them with the available scientific evidence, and selecting among alternative courses of action that best serve the patient's interests (Bhugra et al., 2011). Having long been applied to the study of decision-making, DPT may provide a useful conceptual framework for understanding clinical decision-making (Bhugra et al., 2011; Van Den Berg et al., 2020). From this perspective, clinical decision-making can be viewed as the continuous interaction between fast, intuitive judgments that often operate implicitly and slower, analytical, and reflective reasoning processes. A better understanding of these processes may help clinicians consciously balance intuitive and reflective judgments, recognize potential cognitive pitfalls, and refine their clinical reasoning.

DPT offer important insights into the understanding, teaching, and development of clinical decision-making skills. Consistent with this framework, a qualitative study exploring psychiatrists' clinical decision-making processes found that clinicians relied primarily on intuitive (Type 1) processing in familiar and frequently encountered clinical situations, whereas they engaged in more analytical (Type 2) reasoning under conditions of greater uncertainty (Bhugra et al., 2011). The findings indicated that clinical decision-making is strongly influenced by experience, with more experienced psychiatrists relying more heavily on intuition and previous clinical practice. This suggests that, with increasing clinical experience, reasoning processes that initially require deliberate and analytical processing may gradually become automatized, giving rise to faster pattern-recognition mechanisms. Consistent with this interpretation, a study examining how medical students learned to diagnose abnormalities on chest radiographs reported that increasing diagnostic expertise was associated with reduced prefrontal cortex activation, which may reflect a shift from Type 2 to more automatic Type 1 processing (Rotgans, 2022). In addition, external factors such as time pressure, workload, and high-risk clinical situations have been found to encourage clinicians to rely more on intuitive than analytical decision-making. These findings are consistent with DPT, which propose that Type 1 processing becomes increasingly dominant when cognitive resources are limited.

From a dual-process perspective, clinical decision-making depends not on the isolated operation of Type 1 or Type 2 processes but on their interaction (Pelaccia et al., 2011). Within the combined model, Type 1 processing is thought to rapidly generate diagnostic hypotheses during the early stages of a clinical encounter, whereas Type 2 processing evaluates, confirms, or, when necessary, revises these hypotheses. Because Type 2 reasoning operates on the hypotheses generated by Type 1 processing, intuitive processing influences not only the formation of initial diagnostic impressions but also which diagnostic hypotheses are subsequently considered during analytical reasoning. Clinical decision-making can therefore be understood as a dynamic process shaped by the continuous interaction between intuitive (Type 1) and analytical (Type 2) processing, rather than by the alternating operation of two independent systems.

Research on clinical decision-making within the framework of DPT may also contribute to the debate on rationality. In a five-year follow-up study conducted in a psychosomatic consultation service, intuitive judgments that generated a feeling of inconsistency between the clinician's current diagnosis and the patient's presentation played an important role in correcting diagnostic errors (Seidl and Seidl, 2021). This finding suggests that intuitive processes may function not only as a source of cognitive biases but also, when appropriate, as an early warning signal that triggers analytical re-evaluation. Similarly, an experimental study examining physicians' interpretation of chest X-ray and electrocardiograms under different viewing durations found that high levels of diagnostic accuracy could be achieved even with brief exposure times, whereas extending the evaluation time did not consistently improve diagnostic accuracy (Monteiro et al., 2024). Under some conditions, additional analytical evaluation provided no incremental benefit and was even associated with reduced performance. Taken together, these findings suggest that intuitive judgments should not be regarded solely as a source of cognitive bias responsible for errors and that the contributions of Type 1 and Type 2 processes to clinical reasoning depend on the demands and context of the clinical situation.

One of the most distinctive examples of intuitive clinical judgment in psychiatric practice is the concept of praecox feeling. Originally described by H. C. Rümke, this concept refers to a subtle sense of strangeness, bizarreness, or difficulty establishing rapport that arises during the first few minutes of a clinician's encounter with a schizophrenia patient and is often difficult to articulate verbally (Rümke and Neeleman, 1990). Although praecox feeling is not regarded as a diagnostic criterion, it is viewed as an important component of the clinical decision-making process and as a potential source of the early diagnostic hypotheses formulated by psychiatrists. From a dual-process perspective, praecox feeling may be understood as a Type 1 clinical hypothesis that emerges rapidly and implicitly through the clinician's encounter with the patient. In this sense, it has been argued that praecox feeling should be regarded not as a diagnostic tool but as a diagnostic compass that directs the clinician toward further analytical (Type 2) evaluation (Gozé et al., 2019).

Taken together, from a dual-process perspective, expert clinical judgment involves neither exclusive reliance on intuition nor systematic suppression of intuitive impressions, but the flexible coordination of Type 1 and Type 2 processing according to the demands of the clinical situation. Beyond their contribution to understanding psychopathology and clinical decision-making, DPT may also provide a useful framework for understanding the mechanisms underlying psychotherapeutic change.

### 6.3 Dual-process theories in understanding psychotherapeutic change

Psychotherapies have been practiced for more than a century within diverse theoretical traditions and encompass a wide range of intervention techniques. Nevertheless, the mechanisms through which different psychotherapeutic approaches exert their effects, as well as the common mechanisms of therapeutic change, remain subjects of ongoing debate. DPT may provide a useful conceptual framework for understanding these mechanisms.

From a DPT perspective, psychotherapeutic change can be conceptualized at three complementary levels, reflecting different ways in which therapeutic change may occur. First, many psychotherapeutic approaches primarily enhance reflective processing (Type 2). For example, cognitive-behavioral therapies (CBT) aim to strengthen the regulatory functions of Type 2 processing by helping individuals identify, question, and generate alternative interpretations of negative automatic thoughts (Clark and Beck, 2010). Similarly, mentalization-based approaches seek to enhance reflective functioning by increasing individuals‘ capacity to think about their own and others' mental states (Taylor, 2010).

A second level of psychotherapeutic change involves the reorganization of the relationship between automatic Type 1 and reflective Type 2 processes. In insight-oriented and expressive therapies, relational patterns, defenses, transference reactions, and other experiences that initially operate outside awareness are explored and gradually brought into reflective awareness within the therapeutic relationship. This process allows experiences that were initially processed in an implicit and automatic manner to become increasingly accessible to reflective and symbolic processing. From this perspective, psychotherapy may be understood as facilitating the integration of automatic modes of processing into the domain of reflective functioning (Taylor, 2010). Along similar lines, Wilma Bucci's Multiple Code Theory explains an important aspect of therapeutic change in terms of strengthening the connections between bodily-emotional experience and verbal and symbolic representations (Bucci, 2002). Becoming aware of emotions, labeling them, and linking them with memories, images, and fantasies may facilitate the mental processing and regulation of emotional arousal.

An important implication of this perspective concerns the nature of therapeutic communication. Robbins (2018) argued that attempting to translate bodily, emotional and pre-symbolic experiences prematurely into reflective or interpretive language can disrupt therapeutic communication. When primary process thinking or subsymbolic experience is conceptualized as a qualitatively distinct mode of mental organization, the therapist's task is not to assume that these experiences are already available for reflective interpretation, but to help them become increasingly thinkable and available for symbolic representation. Otherwise, a translational mismatch may arise, whereby the therapist attempts to interpret experiences that the patient is not yet able to represent or reflect upon at that symbolic level (Robbins, 2018).

However, psychotherapeutic change may not be fully explained by strengthening Type 2 processing or reorganizing the relationship between Type 1 and Type 2 processing. A third level of therapeutic change involves more direct modifications of Type 1 processing. A significant proportion of therapeutic change may occur through implicit, emotional, and relational processes (Hyland, 2020). In particular, a safe and emotionally regulating therapeutic relationship may gradually reshape established interpersonal expectations and automatic patterns of emotional responding. The concept of corrective emotional experience, emphasized within relational psychodynamic traditions, can be understood within this framework. Experiencing a relationship that differs from previous interpersonal experiences may contribute not only to the acquisition of new explicit knowledge but also to changes in interpersonal expectations, emotional associations, and automatic response patterns. Such changes are likely to occur primarily through implicit learning rather than the acquisition of declarative knowledge.

Similarly, psychotherapeutic change involves a learning process through which skills that initially require conscious effort—such as identifying and questioning automatic thoughts, generating alternative interpretations, or using new coping strategies in CBT—gradually become more automatic with repeated practice. From a dual-process perspective, these skills may initially rely on reflective processing but, through repeated application, become progressively integrated into associative processing and consolidated over time (Beevers, 2005; Weinberger and Stoycheva, 2019).

Taken together, the effectiveness of psychotherapy cannot be explained solely in terms of bringing unconscious experiences into awareness. More broadly, psychotherapy may be conceptualized as a multilayered process of change involving the reorganization of the relationship between automatic and reflective modes of processing, the enhancement of reflective capacity, and the gradual transformation of deeper associative patterns through learning (Figure 1). Nevertheless, the processes described here should not be regarded as distinct mechanisms separated by clear boundaries. Different psychotherapeutic approaches engage several of these processes simultaneously, with reflective awareness, symbolization, emotional experience, and implicit learning interacting throughout the therapeutic process. Accordingly, the distinctions proposed within a dual-process framework should be viewed not as an exhaustive account of the psychotherapeutic change, but rather as an attempt to facilitate an understanding of the different mechanisms underlying therapeutic change.

## 7 Neurobiological perspective

Having examined DPT from psychodynamic and clinical perspectives, we now turn to the neurobiological evidence relevant to these theories. Numerous studies have investigated the neurobiological basis of the different modes of processing proposed by DPT. A substantial body of this research has focused on the distinction between fast, automatic Type 1 processing and slow, controlled Type 2 processing, using experimental paradigms that require response inhibition. In addition to executive function tasks such as the Stroop, Go/No-Go, and Stop-Signal paradigms, reasoning tasks including belief bias, the Linda problem, and the Cognitive Reflection Test (CRT) have been widely used. Within this framework, Type 1 processes are assumed to generate an initial response, whereas Type 2 processes are recruited to monitor, control, or inhibit this response.

Early neurobiological studies examining this framework focused on the roles of the prefrontal cortex and anterior cingulate cortex (ACC) in cognitive control (Gronchi et al., 2024). According to the classical account, when conflict arises between an automatic intuitive response and a more analytical evaluation, the ACC detects the conflict, after which executive control mechanisms, primarily mediated by the right prefrontal cortex, are recruited to suppress the intuitive response (Evans and Stanovich, 2013). Consistent with this account, studies using paradigms such as belief bias and the CRT, in which Type 1 and Type 2 processes generate competing responses, have shown that conflict is associated with increased ACC activation, whereas successful analytical responding is accompanied by activation of the right inferior frontal gyrus (r-IFG); (De Neys et al., 2008; Tsujii et al., 2011). These findings appear to support the classical dual-process account, according to which automatically generated intuitive responses are suppressed by executive control mechanisms.

However, subsequent studies have suggested that this inhibition model may be insufficient. For example, some studies have reported that performance on the CRT is more strongly associated with activation of the posterior cingulate cortex (PCC), a key hub of the Default Mode Network (DMN), than with executive control networks (Vartanian et al., 2018). The DMN, which includes the medial prefrontal cortex, inferior parietal lobule (IPL), lateral temporal cortex, and hippocampus, has been associated with resting-state cognition and mind wandering. Accordingly, some researchers have proposed that the DMN may represent one of the neural substrates underlying Type 1 processing, functioning as a fast and spontaneous “autopilot” system (Gronchi and Giovannelli, 2018). However, the association between performance on the CRT—regarded as an indicator of Type 2 processing—and DMN activation suggests that reflective thinking may involve processes beyond inhibition and executive control.

Large-scale meta-analytic evidence supports this complex view. The meta-analysis conducted by Gronchi et al. (2024) demonstrated consistent activation of regions associated with cognitive control and conflict monitoring—including the medial and superior frontal cortex, the ACC, the insula, and the left inferior frontal gyrus—across various dual-process paradigms. However, these activations were not specific to either Type 1 or Type 2 processing. Therefore, the study did not provide evidence for distinct anatomical regions dedicated to each processing mode. Accordingly, current perspectives emphasize that the systems proposed by DPT should not be regarded as separate anatomical structures with one-to-one counterparts in the brain, but as functional patterns that emerge from the dynamic interaction of multiple neural networks (Gronchi et al., 2024).

Studies investigating the neurobiological basis of DPT are not limited to conflict monitoring and response inhibition paradigms. Researchers have examined the different modes of processing in the context of creativity and problem solving. In this literature, divergent thinking and insight, two key components of creative cognition, have been conceptualized from a dual-process perspective. Divergent thinking, which involves generating multiple solutions to open-ended problems, has been proposed to rely primarily on Type 1 processing, characterized by associative, automatic, and relatively effortless processing. In contrast, insight, characterized by the sudden realization of a solution to a closed-ended problem, has been linked to Type 2 processing involving sustained attention, cognitive control, and the restructuring of mental representations (Kuang et al., 2022). Within this framework, a neuroimaging meta-analysis found that divergent thinking was primarily associated with activation of the IPL, cuneus, and middle frontal gyrus, whereas insight was associated with activation of the IFG, parahippocampal gyrus, amygdala, and superior parietal lobule (Kuang et al., 2022). Comparative analyses showed that divergent thinking was more strongly associated with activation of the IPL, a key hub of the DMN, whereas insight preferentially engaged frontal, parietal, and limbic regions implicated in executive control, attention allocation, and emotional processing (Kuang et al., 2022).

As discussed earlier, some researchers have argued that Type 1 processing is closely linked to bodily and emotional signals, reflecting a more embodied mode of cognition (Bellini-Leite, 2022). One of the most influential neurobiological accounts consistent with this perspective is Damasio's Somatic Marker Hypothesis (Bechara and Damasio, 2005). According to this model, decision-making is not solely the product of conscious reasoning; bodily and emotional signals associated with previous experiences also play an important role in evaluating behavioral alternatives. Physiological and emotional responses that accompanied similar situations in the past can be rapidly reactivated when comparable situations are encountered, guiding behavior before conscious evaluation begins. Damasio proposed that structures such as the brainstem, hypothalamus, insula, and somatosensory cortex contribute to decision-making by representing these bodily states at the neural level.

Within this framework, somatic markers have been proposed to function as emotional signals that guide decision-making before conscious deliberation. One of the strongest experimental findings supporting this hypothesis comes from the Iowa Gambling Task. In a study, healthy individuals developed anticipatory physiological responses to disadvantageous options before they were consciously able to distinguish advantageous from disadvantageous choices (Chick, 2019). In contrast, individuals with ventromedial prefrontal cortex lesions failed to develop these responses and showed impaired decision-making performance (Chick, 2019). These findings suggest that bodily states contribute to intuitive decision-making before conscious evaluation, supporting an embodied view of Type 1 processing.

An exhaustive discussion of the neurobiological mechanisms underlying different modes of cognitive processing is beyond the scope of this review. Nevertheless, current evidence broadly suggests that distinct cognitive processes are supported by different brain networks operating in dynamic interaction. At the same time, given that DPT is primarily a psychological framework, these findings should not be interpreted as demonstrating direct neurobiological counterparts of Type 1 and Type 2 processes (Gronchi et al., 2024). Rather, psychological theories and neurobiological accounts address mental phenomena at different levels of explanation, and the relationship between them is better understood as one of complementarity rather than direct correspondence.

## 8 Discussion and conclusion

Understanding the complexity of the human mind has been a longstanding scientific and intellectual endeavor, from the philosophical debates of Ancient Greece to psychoanalytic theory, experimental psychology and contemporary neuroscience. DPT have made an important contribution to this effort by viewing the mind not as a single, homogeneous system but as a multilayered organization arising from the interaction of different modes of processing. However, fundamental questions about the nature of these processes, how they are organized, and how they interact remain unresolved.

Psychodynamic theories have long sought to explain the organization of mental processes and developed the concept of primary process thinking to explain how conscious and unconscious mental processes are organized. Based on the perspectives reviewed in this article, primary process thinking can be conceptualized in two ways: either as a qualitatively distinct mode of processing from conscious thought or as a less differentiated form of the same underlying mental organization. Although these theories were not developed within DPT, they provide valuable insights into the nature of different modes of mental processing and offer an alternative perspective from which distinctions proposed by DPT can be reconsidered. These debates therefore extend beyond the nature of primary process thinking itself and raise broader questions about how the architecture of the mind should be conceptualized.

In addition, applying different perspectives within DPT to psychopathology, clinical decision-making, and psychotherapeutic change illustrates that dialogue between theoretical traditions can be productive not only conceptually but also clinically. Likewise, findings from neuroscience suggest that the distinctions proposed by DPT should not be reduced to individual brain regions but instead understood in terms of the dynamic interaction of distributed neural networks.

As demonstrated throughout this article, one of the main contributions of DPT may be that it provides a common conceptual language for examining the relationships between conscious and unconscious, intuitive and reflective, embodied and symbolic, and automatic and controlled processes. Accordingly, in this review, DPT has been considered neither as a comprehensive theory of mental life nor as a narrow model limited to experimental psychology. Rather, it has been used as a conceptual framework to bring psychodynamic theories, clinical observations, and neurobiological findings into a common discussion. However, several methodological and conceptual limitations should be acknowledged. This article presents a conceptual and interdisciplinary synthesis rather than a systematic review. Conceptually, integrating different fields may overlook some field-specific aspects. For example, cognitive reformulations of primary process thinking have been criticized for deemphasizing motivational and conflict-related aspects central to psychoanalytic theory (Gammelgaard, 2006). Additionally, comparing concepts across different levels of explanation may risk a category mistake if their similarities are taken to imply conceptual equivalence (Ryle, 2009). Accordingly, bringing DPT into dialogue with psychodynamic, clinical, and neurobiological perspectives does not imply that their constructs are directly interchangeable.

Taking these limitations into account, several points should be considered when linking DPT with other fields. First, as illustrated by the discussion of primary and secondary process thinking, the nature of the conceptual bridge may depend on how the relevant mental process is conceptualized. Interdisciplinary dialogue therefore requires careful examination of the features or mechanisms underlying these parallels. In particular, applying Type 1/Type 2 terminology to psychodynamic and clinical phenomena requires caution because DPT comprises a family of accounts emphasizing different processing characteristics rather than universally shared properties. Similarly, clinical conditions or psychotherapeutic approaches should not be classified as inherently Type 1 or Type 2, as multiple mechanisms may coexist and interact within the same clinical phenomenon or therapeutic process. Finally, as noted above, differences in explanatory levels should be considered when drawing parallels between DPT and other theoretical, clinical, or neurobiological accounts. Within these boundaries, DPT may provide a useful framework for examining how different accounts of mental functioning converge and diverge.

These considerations also point to several directions for future research. Future studies should begin by clearly defining the processes under investigation and then derive testable hypotheses from these conceptual distinctions. One question arising from this review is whether different modes of processing are best understood as qualitatively distinct or as varying along a continuum. Experimental, developmental, and clinical studies could examine whether processing characteristics vary categorically or gradually across task demands, levels of awareness, cognitive control, developmental stages, or clinical conditions. Research should also investigate whether distinct representational modes and graded differences in differentiation can coexist and interact rather than represent competing alternatives. In psychopathology and psychotherapy, attention should be given to how different processing mechanisms interact and how their relationships change with symptom development and therapeutic change. Addressing these questions through interdisciplinary research designs combining psychodynamic concepts and clinical observations with methods from cognitive psychology and neuroscience could move the field from broad conceptual parallels toward precise, empirically testable accounts of mental functioning.
